# Health workforce governance during the COVID-19 pandemic: learning lessons from Europe: Gemma Williams

**DOI:** 10.1093/eurpub/ckac129.041

**Published:** 2022-10-25

**Authors:** G Williams, J Buchan, T Zapata

**Affiliations:** 1 European Observatory on Health Systems and Policies, London, UK; 2 University of Edinburgh, Edinburgh, UK; 3 Health Foundation, London, UK; 4 WHO Regional Office for Europe, Copenhagen, Denmark

## Abstract

**Background:**

This study considers some of the effective governance tools that have been utilised to mobilise, redeploy and repurpose the health workforce during the COVID-19 pandemic to create surge capacity, protect workforce health and wellbeing and ensure effective implementation of vaccination programmes.

**Methods:**

Data were systematically extracted from the Observatory/WHO Europe/European Commission Health System and Response Monitor, covering the period from March 2020 to May 2021 with a focus on four dimensions of health workforce governance: national/regional government policies; legislation; regulation; the role and remit of employers and management.

**Results:**

A wide-range of governance actions across all levels were required to ensure the health workforce could provide effective pandemic responses. Creating surge capacity, for example, often required adoption of emergency legislation to facilitate exceptional hiring procedures and the changing of (re-)registration requirements, as well as additional training and development of new competencies among other actions. Putting in place physical and mental health support meanwhile required defining infection control policies, monitoring PPE supply and distribution, ensuring access to free mental health support, and implementation of breaks. Some countries also allowed “new” types of workers to vaccinate; online or in person training; adjustments to payment mechanisms; and creating new supervision requirements.

**Conclusions:**

Pandemic responses have broken up sclerotic governance structures which have hampered past health workforce development and reform, new training programmes have been rapidly developed, leadership roles have been delegated to a wider-range of health professionals than before and monitoring systems that provide more rapid data on staffing levels have been put into place. Learning from and evaluating these changes will be important to help inform future pandemic responses.

